# Elucidation of Clathrin-Mediated Endocytosis in *Tetrahymena* Reveals an Evolutionarily Convergent Recruitment of Dynamin

**DOI:** 10.1371/journal.pgen.0010052

**Published:** 2005-11-04

**Authors:** Nels C Elde, Garry Morgan, Mark Winey, Linda Sperling, Aaron P Turkewitz

**Affiliations:** 1 Department of Molecular Genetics and Cell Biology, University of Chicago, Chicago, Illinois, United States of America; 2 Department of Molecular, Cellular, and Developmental Biology, University of Colorado, Boulder, Colorado, United States of America; 3 Centre de Genetique Moleculaire, Centre National de la Recherche Scientifique, Gif-sur-Yvette, France; Washington University, United States of America

## Abstract

Ciliates, although single-celled organisms, contain numerous subcellular structures and pathways usually associated with metazoans. How this cell biological complexity relates to the evolution of molecular elements is unclear, because features in these cells have been defined mainly at the morphological level. Among these ciliate features are structures resembling clathrin-coated, endocytic pits associated with plasma membrane invaginations called parasomal sacs. The combination of genome-wide sequencing in *Tetrahymena thermophila* with tools for gene expression and replacement has allowed us to examine this pathway in detail. Here we demonstrate that parasomal sacs are sites of clathrin-dependent endocytosis and that AP-2 localizes to these sites. Unexpectedly, endocytosis in *Tetrahymena* also involves a protein in the dynamin family, Drp1p (Dynamin-related protein 1). While phylogenetic analysis of AP subunits indicates a primitive origin for clathrin-mediated endocytosis, similar analysis of dynamin-related proteins suggests, strikingly, that the recruitment of dynamin-family proteins to the endocytic pathway occurred independently during the course of the ciliate and metazoan radiations. Consistent with this, our functional analysis suggests that the precise roles of dynamins in endocytosis, as well as the mechanisms of targeting, differ in metazoans and ciliates.

## Synopsis

The wings of bats and of birds are similar structures with similar functions but nonetheless evolved independently within these two different branches of animals. Many examples of this phenomenon, called convergent evolution, are known at the level of whole organisms. Here, the authors demonstrate that convergent evolution has also occurred at the level of individual cells, in a pathway responsible for taking up membrane from the cell surface. The authors took advantage of the recent genomic sequencing of distantly related organisms, and in particular of the single-celled ciliate *Tetrahymena thermophila*. In animal cells, one of the proteins required for membrane uptake is called dynamin. Dynamin is not required for this function in most nonanimal cells, but the authors discovered that *Tetrahymena* is an exception and that it uses a close relative of dynamin for particle uptake. After reconstructing the history of dynamin proteins, the authors found that the specific role in membrane uptake evolved independently in *Tetrahymena* and in animals.

## Introduction

**Figure 1:**
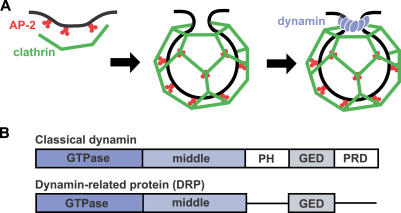
Overview of Endocytosis (A) Schematic diagram of clathrin-coated vesicle formation in metazoans. AP-2 (red) serves as an adapter. It can interact with receptors destined for internalization while also recruiting clathrin (green) to the plasma membrane. Clathrin assembly at those sites drives or facilitates membrane invagination. Dynamin (blue) assembles at the neck of a nascent vesicle to promote membrane fission. (B) Domains of classical dynamin and of DRPs.

Endocytosis is conserved in eukaryotes, but the molecular machinery deployed by cells to internalize plasma membrane varies according to task, which can range from nutrient absorption to cell signaling [1]. The best understood mechanism of endocytosis involves clathrin-induced membrane deformation to form nascent vesicles (Figure 1A) [2]. Clathrin can also recruit membrane proteins for internalization, often via a multimeric adaptor protein (AP) complex, AP-2 [3]. In metazoans, clathrin-mediated endocytosis (CME) requires classical dynamin, a member of a family of self-assembling GTPases. During endocytosis, the GTP-dependent constriction of an oligomeric dynamin “collar” may induce fission of vesicles from plasma membrane [4,5]. Additionally, dynamin recruits effectors, including actin-binding proteins, which could mediate aspects of vesiculation [6,7].

Processes closely resembling CME have been described in a small number of eukaryotes outside Metazoa, but it is not clear that any depend on proteins in the dynamin family (dynamin-related proteins [DRPs]). In *Saccharomyces cerevisiae,* endocytic vesicle formation involves some proteins associated with CME [8,9], but no endocytic role has been discovered for AP-2 or any DRP [10,11]. A wide survey of eukaryotes suggests that the most conserved role for DRPs is in mitochondrial inheritance, while other family members mediate membrane remodeling events distinct from endocytosis [12,13]. For example, while *Trypanosoma brucei* uses CME for turnover of surface glycoproteins [14], its single DRP is dedicated to mitochondrial fission [15]. Another eukaryote deeply divergent from metazoans, the red alga *Cyanidioschyzon merolae,* has two DRPs, one acting in mitochondrial fission and the other in chloroplast division [16,17].

Classical dynamins are composed of five domains (see Figure 1B). Three of these are found in all DRPs: a large N-terminal GTPase domain, a middle domain, and a GTPase effector domain (GED) [13]. The remaining two domains in classical dynamin, pleckstrin homology (PH) and proline-rich domain (PRD), are implicated in targeting to endocytic pits and in the recruitment of SH3 domain-containing proteins, respectively, which in turn regulate actin assembly at these sites [18,19]. These observations are consistent with a recent expansion of the role of DRPs to include CME, in an evolutionary step involving the acquisition of PH domains and PRDs. Classical dynamins are exclusive to Metazoa with two known exceptions, *DRP2A* and *DRP2B,* in *Arabidopsis thaliana.* These nearly identical genes are highly diverged from other dynamins and may be involved in vesiculation of the trans-Golgi network [20,21].

These observations support the view that the founding member of the dynamin family was a DRP required for maintenance of a mitochondrial endosymbiont [12]. Subsequently, gene duplication and differentiation led to the acquisition of new functions. We examined the large radiation of DRPs within ciliates and found that these proteins encompass a surprising variety of roles. In conjunction, we performed molecular characterization of CME in *Tetrahymena,* along with phylogenetic analysis of components in this pathway. This combination provided insight into the evolution of both endocytosis and dynamin that would not have been evident by taking either approach alone.

## Results

### Coat-Mediated Endocytosis Visualized with FM1–43 in *Tetrahymena*

**Figure 2:**
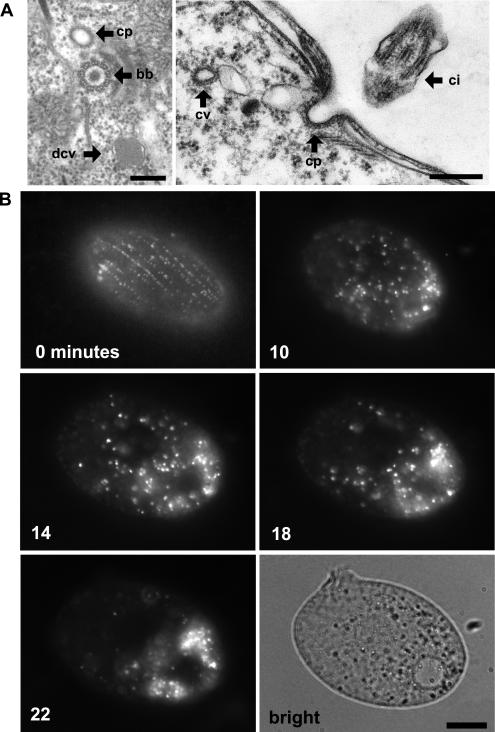
Visualizing Sites of Endocytosis (A) In *Tetrahymena,* coated pits (cp) are found near the base of cilia, as shown in tangential (left) and cross (right) sections. bb, ciliary basal body; ci, cilia; cv, coated vesicle; mt, mitochondrion; dcv, dense core vesicle. Bars = 200 nm. (B) Time course of FM1–43 dye uptake. A cell shown immediately after treatment with 5 μM FM1–43 (0 min) shows rows of fluorescent puncta at the cell surface. Time-lapse images (10, 14, 18, and 22 min) of a single cell following 5-min exposure to FM1–43. At the later time points (18 and 22 min), the dye accumulates in what appear as vesicles clustered toward the cell posterior. The brightfield image shows the cell at the end of the time course. Bar = 10 μm.

**Figure 3:**
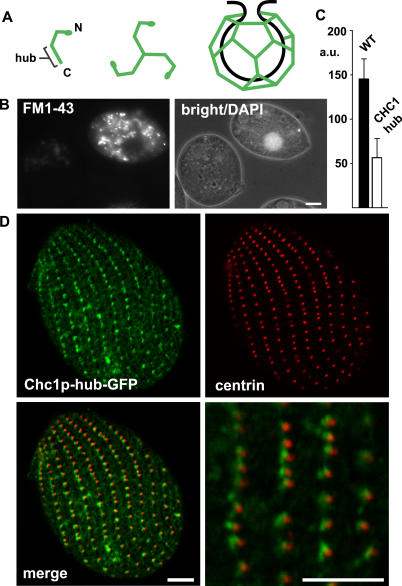
Analysis of CHC (A) Cartoon of CHC assembly. The location of the C-terminal hub domain is indicated. (B) Expression of truncated CHC (hub) inhibits endocytosis. Wild-type (DAPI-labeled) and CHC hub-expressing cells were mixed and incubated with FM1–43. Reduced FM1–43 uptake is detected in the hub-expressing cell. (C) Quantification of FM1–43 uptake. FM1–43 uptake was quantified by analysis of 20 image pairs such as those shown in (B), as described in Materials and Methods. The fluorescence units are arbitrary (a.u.). WT, wild-type. (D) Cells expressing Chc1p-hub-GFP (from the *MTT1* promoter) were fixed, permeabilized, and labeled with anti-centrin antibody. The merged image shows the close proximity of clathrin hub-GFP to basal bodies, comparable to Figure 2. Bar = 5 μm (B and D).

As previously noted, electron microscopy of *Tetrahymena* and *Paramecium* thin sections reveals structures, situated near ciliary basal bodies, that resemble coated pits in mammalian cells (Figure 2A) [22,23]. We set out to observe the activity of these structures in live cells. No endocytic cargo molecules in *Tetrahymena* are known, but endocytic vesicles in a variety of species have been vitally stained using the styryl dye FM1–43 [24]. To preclude FM1–43 uptake via phagocytosis at the oral apparatus, we starved cells for 2 h prior to FM1–43 labeling, a treatment that temporarily eliminates phagosome formation via the regression and eventual replacement of the preexisting oral apparatus [25]. Exposing such starved cultures to FM1–43 led to the immediate appearance of fluorescent puncta, aligned in rows, near the cell surface (Figure 2, 0 min). This pattern recalled the known arrangement of ciliary basal bodies (Figure 3, centrin), with their associated coated pits (see Figure 2A). To observe the itinerary of the putative endocytic vesicles, cultures were exposed to dye for 5 min, and individual cells were observed over time. Ten minutes after exposure to dye, the putative vesicles appeared highly mobile throughout the cytoplasm (Figure 2, 10 min). Over the next 20 min, they appeared to coalesce and accumulate in the posterior of the cell (Figure 2, 14 and 18 min). At the end of this period, the majority of puncta appeared to have coalesced (Figure 2, 22 min and brightfield). Therefore, kinetic analysis of FM1–43 uptake resembled the expected pattern of an endocytic pathway originating from coated pits observed near basal bodies and suggested the existence of a localized endosomal compartment.

### Clathrin Is Essential for FM1–43 Uptake

To determine whether FM1–43-labeled vesicles specifically reflected CME in this system, we identified a single clathrin heavy chain (CHC) ortholog *(CHC1)* in the *Tetrahymena* macronuclear genome. *CHC1* encodes a predicted protein of 1,710 residues, 40% identical to bovine clathrin. In mammalian cells, clathrin function can be blocked in a dominant negative fashion by expressing the C-terminal third of the protein, called the hub (see Figure 3A) [26]. We cloned the *CHC1* hub, placing it under the control of the cadmium-inducible *MTT1* promoter, and used this to transform *Tetrahymena* [27]. Within 3 h of cadmium addition, FM1–43 uptake was blocked (Figure 3B and 3C). In the absence of cadmium, internalization of FM1–43 by these cells was indistinguishable from that of wild-type. Cadmium addition itself did not block FM1–43 uptake, in either wild-type cells or cells in which the *CHC1* hub was replaced with *GRL1,* a constituent of secretory granules [28] (data not shown). These results suggest that FM1–43 selectively labels vesicles derived by CME in *Tetrahymena*.

The Chc1p hub, tagged with green fluorescent protein (GFP), localized at the plasma membrane in an ordered array. Moreover, dual labeling with an antibody against centrin demonstrated that it was targeted near basal bodies of cilia (Figure 3D). This is consistent with the location of coated pits (see Figure 2A). Taken together, the data support the idea that uptake of FM1–43 can be directly blocked by the Chc1p hub acting at coated pits.

### A Family of Adaptor Proteins in *Tetrahymena*

**Figure 4:**
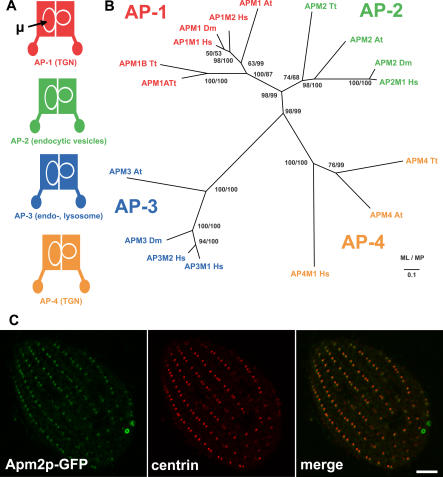
Phylogenetic Analysis and Localization of the AP-2 μ Subunit (A) Schematic representation and cellular localization of the heterotetrameric AP complexes. Location of the μ subunit is indicated. TGN, trans-Golgi network. (B) AP complex μ subunit phylogeny. The topology illustrated is the best maximum likelihood (ML) distance for μ subunits from *A. thaliana* (At), *D. melanogaster* (Dm), *H. sapiens* (Hs), and *T. thermophila* (Tt). Both ML and maximum parsimony (MP) bootstrap support values are shown at each node. (C) Fixed, permeabilized cells expressing GFP-tagged AP-2 μ subunit (Apm2p-GFP) (from the *MTT1* promoter) were labeled with anti-centrin antibody. The distribution of Apm2p-GFP is similar to that of the CHC hub (Figure 3), but the former is additionally present at the contractile vacuole pores. Bar = 5 μm.

Endocytosis in animal cells involves AP-2, one of a family of heterotetrameric complexes that mediate diverse membrane trafficking events involving clathrin in many eukaryotes (Figure 4A) [3]. In *Tetrahymena,* we identified four paralogs encoding AP medium subunits (μ subunits). Phylogenetic analysis of μ subunits, including *Tetrahymena,* several metazoans, and *Arabidopsis,* produced a topology in which subunits distributed into functional groups across species (Figure 4B). This tree supports an early diversification of μ subunits, consistent with prior analysis [29,30], and specifically suggests an origin of adaptor-mediated functions that predates the split between ancestors of metazoans and ciliates. Of the *Tetrahymena* μ subunit genes, a single paralog clustered with the AP-2 family, while two paralogs *(APM1A* and *APM1B)* clustered in the AP-1 family. Interestingly, this organism has no recognizable subunits of AP-3, a complex found in a variety of eukaryotes, while the genome does appear to include genes for AP-4 subunits, a complex found previously only in a subset of metazoans and plants [29]. Although not shown, the genome contains the expected large and small AP subunits that compose each of three heterotetrameric complexes.

To determine if the phylogenetic classification of the *Tetrahymena* subunits was consistent with their sites of action, we cloned and GFP-tagged *APM1A, APM1B,* and *APM2.* Apm2p-GFP localized near sites of endocytosis in a pattern similar to that of clathrin (Figure 4C). Uniquely among the proteins included in this study, Apm2p-GFP labeled two ring structures in the posterior of the cell, the contractile vacuole pores, indicating a possibly novel function for this protein. GFP-tagged Apm1Ap and Apm1Bp both localized to internal structures in the cell (not shown). The presence of Apm2p-GFP at sites of CME reinforces the roles suggested by the molecular conservation in this pathway. We did not investigate potential effects of *APM2* knockdown, because the AP-2 complex appears unlikely to be required for the receptor-independent uptake of FM1–43.

### A Dynamin-Related Protein Is Required for Clathrin-Mediated Endocytosis

**Figure 5:**
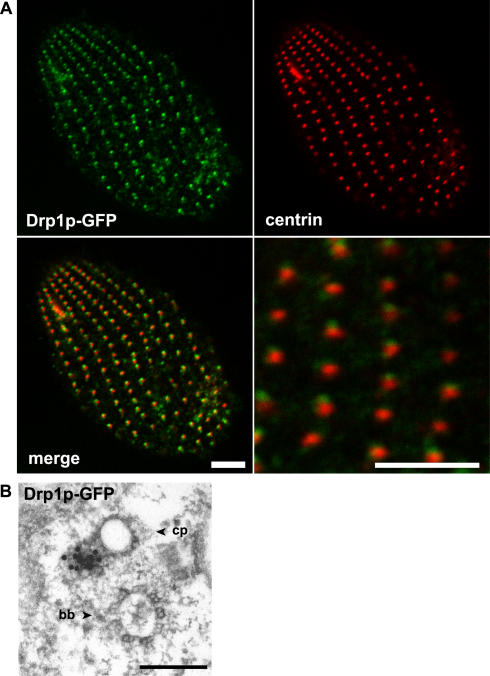
Localization of Drp1p (A) Fixed, permeabilized cells expressing Drp1p-GFP (from the *MTT1* promoter) were labeled with anti-centrin antibody. Like CHC and AP-2 μ subunit, Drp1p-GFP localizes to sites near basal bodies. Bars = 5 μm. (B) Immuno-gold visualization of Drp1p-GFP shows localization to a coated pit (cp) near a basal body (bb). Bar = 200 nm.

DRPs have not been clearly demonstrated to participate in endocytosis except in metazoans, but the presence of an unusually large number of DRPs in the *Tetrahymena* genome prompted us to ask if any might contribute to CME. Drp1p lacks both the PH domain and PRD of classical dynamins. The GTPase domain of Drp1p is 47% identical to human dynamin-1, while the middle domain and GED are 30% and 27% identical to dynamin-1, respectively. When *DRP1*-*GFP* was expressed in wild-type cells, it nearly co-localized with centrin, consistent with the location of Chc1p hub-GFP just anterior to basal bodies (Figure 5A). Further examination of Drp1p-GFP localization by immunoelectron microscopy of cryofixed cells confirmed a close association of Drp1p-GFP with coated pits (Figure 5B). Gold labeling appears adjacent to the coated pits, but this may not mirror the precise distribution of endogenous Drp1p at these sites, because GFP-tagging itself may interfere with the activity of Drp1p, as it does for other dynamin family proteins in other systems.

**Figure 6:**
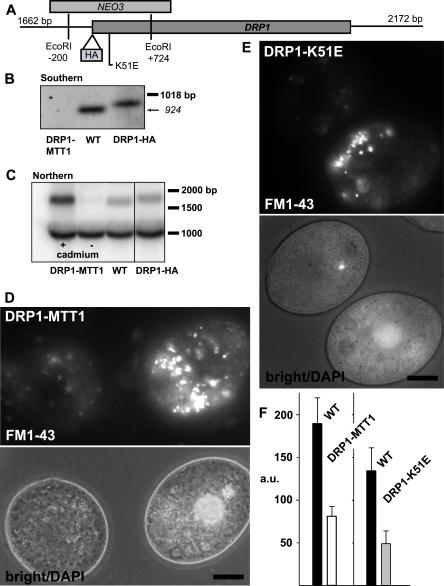
Functional Analysis of *DRP1* (A) The *DRP1* locus, with strategy for *NEO3* disruption and HA epitope tagging, both via homologous recombination. (B) Southern blot of wild-type (WT) and *drp1–1::neo3* lines rescued with *DRP1* integrated at the *MTT1* locus (DRP1-MTT1) or *DRP1* tagged with HA (DRP1-HA). (C) Northern blots of wild-type (WT) and rescued (DRP1-MTT1) lines. Analysis of *DRP1-MTT1* maintained with (+) or without (−) cadmium for 16 h demonstrates that transcript expression is cadmium-dependent. The expression of *DRP1-HA,* at the endogenous locus, is comparable to wild-type. The band near 1 kb serves as a loading control. (D) FM1–43 uptake depends on Drp1p. *DRP1-MTT1*cells were maintained in cadmium-free medium for 16 h and mixed with wild-type (DAPI labeled) as described in text. (E) Impaired uptake of FM1–43 in cells expressing, from the *MTT1* promoter, the *K51E* allele of *DRP1*. (F) Quantitative comparison of FM1–43 uptake, shown in arbitrary fluorescence units (a.u.). Bar = 10 μm (D and E).

**Figure 7:**
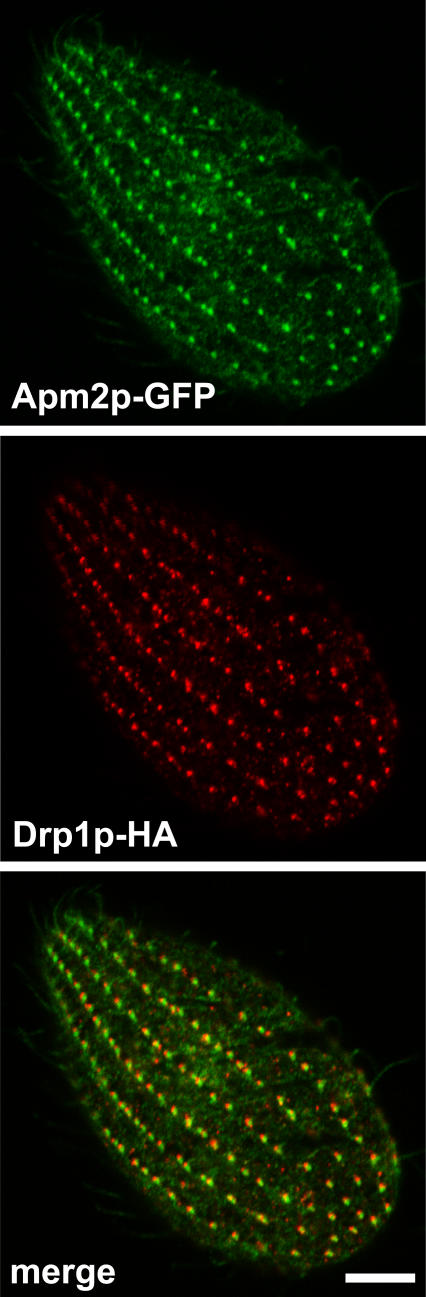
Dual Localization of Drp1p-HA and Apm2p-GFP Cells expressing Drp1p-HA at the wild-type locus were transformed to express Apm2p-GFP driven by the *MTT1* promoter. Fixed, permeabilized cells were immunolabeled with anti-HA antibody. The merged image (bottom) indicates that the two proteins are present in adjacent, partially overlapping puncta. Bar = 5 μm.

To study the effect of depleting Drp1p from cells, we targeted the transcriptionally silent, germline nucleus of *Tetrahymena* for disruption of *DRP1.* We thus obtained heterokaryon strains, which still bore intact copies of *DRP1* in the transcriptionally active macronucleus. When mated, the progeny of these strains lose all intact copies of *DRP1* as a consequence of nuclear remodeling [31]. Such progeny were unable to divide beyond two or three generations, indicating that the gene was essential for growth. To confirm that the defect was due to disruption of *DRP1,* a strain was derived from the Δ*DRP1* cells by rescuing the progeny of heterokaryon matings with full-length *DRP1* tagged with the HA epitope. Southern analysis of a strain expressing *DRP1-HA* confirmed the complete replacement of wild-type *DRP1* with the HA-tagged allele (Figure 6). This strain expressed *DRP1-HA* at wild-type levels (see Figure 6C) and grew normally. Importantly, immunofluorescence using an antibody to the HA epitope confirmed the localization of Drp1p to endocytic sites (Figure 7). Co-expression of Apm2p-GFP in this strain confirmed that Drp1p and Apm2p show close, partially overlapping localization (Figure 7). Drp1p and Apm2p are present either at adjacent zones on the same structure or on two adjacent structures (see Discussion for further comment on the localization of AP-2).

To test if *DRP1* is specifically required for endocytosis, we rescued the progeny of Δ*DRP1* heterokaryons by transforming with an intact *DRP1* gene that integrated adjacent to the endogenous *MTT1* promoter. The resulting strain, lacking *DRP1* at its native locus (labeled as DRP1-MTT1 in Figure 6B), depended on cadmium for *DRP1* expression (Figure 6C) and for normal growth, consistent with the Δ*DRP1* phenotype. Removing these cells from cadmium for 16 h resulted in a dramatic reduction in the uptake of FM1–43 compared to wild-type cells (Figure 6D and 6F). Importantly, cells could be maintained, although not dividing, without cadmium for 96 h and then restored to normal growth by replenishing the medium with cadmium. This indicates that the endocytosis phenotype was not the result of a nonreversible, ill effect on cells depleted of Drp1p. Additionally, other subcellular features such as mitochondria retained a wild-type appearance in electron micrographs of cells depleted of cadmium for 16 h (not shown). Inhibition of FM1–43 uptake in cadmium-depleted cells supports a specific requirement for *DRP1* in CME.

Several alleles with single residue substitutions disrupt GTP turnover and impair dynamin function, in a dominant negative fashion, in mammalian cells [32]. These were used to design similar alleles of *DRP1,* taking advantage of the conservation of residues in the tripartite GTP-binding motifs. *Tetrahymena* were very sensitive to the expression of these alleles under the inducible *MTT1* promoter, even in the absence of cadmium. We did not obtain transformants with a Drp1p-T72F construct (analogous to T65F) in multiple attempts. However, cells expressing Drp1p-K51E (analogous to K44E) were recovered. In the presence of cadmium, this strain was impaired for uptake of FM1–43 when compared to wild-type cells (Figure 6E and 6F). The dominant negative activity of this allele confirms an endocytic role of Drp1p and is consistent with its activity as a GTPase.

### *DRP1* Is Not Closely Related to Classical Endocytic Dynamin

**Figure 8:**
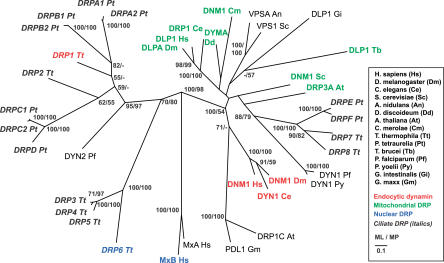
Phylogenetic Analysis of Dynamin and DRPs Best maximum likelihood topology for dynamin and DRPs. Maximum likelihood (ML) and maximum parsimony (MP) bootstrap values over 50% are indicated. Ciliate DRPs *(Tetrahymena* and *Paramecium)* are in italics, DRPs with mitochondrial function are green, nuclear DRPs are in blue, and endocytic dynamins are red. *A. nidulans, Aspergillus nidulans; G. intestinalis, Giardia intestinalis; G. maxx, Glycine max; P. falciparum, Plasmodium falciparum; P. yoelii, Plasmodium yoelii.*

Because DRPs have not previously been clearly identified with roles in endocytosis, we included *DRP1* in phylogenetic analyses to determine if it would associate with classical endocytic dynamins or instead with other classes of DRPs. Phylogenetic comparisons of dynamin and DRPs have revealed a general distribution of proteins according to function, e.g., mitochondrial fission versus vesicle scission [15,33,34]. To determine if this extended to ciliates, we included seven additional DRPs in the *Tetrahymena* genome and nine from a second available ciliate genome, that of *Paramecium tetraurelia,* together with dynamin and DRPs from other eukaryotes. We initially focused our analysis on unambiguously aligned regions in the GTPase domain, middle domain, and GED common to all dynamins and DRPs. (For domain boundary definitions, see Materials and Methods and Figure S1.) The phylogeny generally confirmed a clustering of proteins according to function, as expected (Figure 8). This was especially clear for the classical endocytic dynamins in metazoans and for DRPs implicated in mitochondrial fission across a broad swath of species, including some likely to play this role in ciliates. The localization of *T. thermophila* Drp7p to subcortical mitochondria is consistent with observations of mitochondrial DRP localization in yeast and mammals, supporting its inclusion in the mitochondrial cluster (Figure S2) [12]. In striking contrast, other DRPs from ciliates did not partition into known functional clades but instead appeared to associate among themselves. In particular, *DRP1* is more closely related to other ciliate DRPs than it is to classical metazoan dynamins. Importantly, this conclusion does not depend on the absence of a PRD or PH domain in *DRP1,* because only universally conserved regions of the protein were included in the analysis, nor does it depend on any assumption about the function of the ancestral DRP. This conclusion was also strongly supported in a phylogeny derived by Bayesian analysis of these proteins (Figure S3) and by both maximum likelihood and parsimony-based phylogenies derived by comparison of the GTPase domain, middle domain, or GED alone (not shown). This strongly argues that Drp1p specialized as an endocytic protein after progenitors of ciliates branched from other eukaryotes. Rather than reflecting shared ancestry, the similarity between the roles of Drp1p and classical dynamins is likely to have arisen via functional convergence within this protein family.

### Unique Features of *DRP1*-Mediated Endocytosis

**Figure 9:**
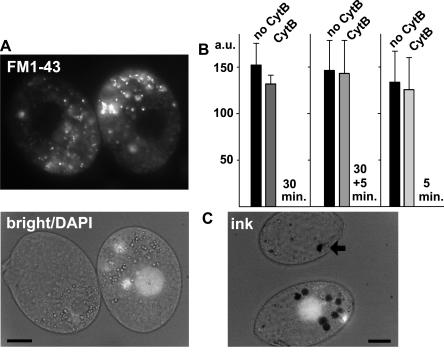
Comparison of the Actin-Dependence of Endocytosis versus Phagocytosis (A) Cells treated with cytB (25 μM, 30 min) were mixed with untreated cells and incubated with FM1–43 (5 min, no cytB). Prior to mixing, cells not treated with cytB were incubated for 10 min with DAPI, to label the nuclei. All cells showed equivalent FM1–43 uptake. (B) Quantitative comparison of FM1–43 uptake, in arbitrary fluorescence units (a.u.). The first set of bars represents pairs (*n* = 4) of cells as described in (A). The second set of bars represents pairs (*n* = 20) of cells in which cytB was also included during the 5-min incubation with FM1–43, to rule out potential reversal of inhibition during drug washout. Exposure to cytB for 5 min did not itself inhibit FM1–43 uptake. This is shown in the third set of bars, representing pairs (*n* = 20) of cells incubated for 5 min in FM1–43 or FM1–43 plus cytB. (C) Under the same conditions as (A), cytB treatment inhibited india ink uptake via phagocytosis from the oral apparatus. Treated cells show a single ink-containing vacuole (arrow), while untreated cells (DAPI labeled) always contain several. Bars = 10 μm.

Classical dynamin can recruit mediators of actin assembly via its C-terminal PRD, and actin assembly appears to be important for endocytosis in some, but not all, animal cells [35]. Actin assembly also appears to be essential for endocytosis in a variety of unicellular organisms [36,37]. *Tetrahymena* Drp1p is missing the PRD but could associate with actin via a PRD-independent mechanism [38]. We first asked whether actin assembly was indeed required for endocytosis in this lineage, by measuring FM1–43 uptake following treatment with chemical inhibitors of actin assembly. *Tetrahymena* treated for 30 min with cytochalasin B (cytB; 25 μM) showed no inhibition of FM1–43 uptake compared to wild-type cells (Figure 9A and 9B). To confirm that this concentration of cytB blocked a bona fide actin-dependent process, we monitored uptake of ink particles via phagosome formation. As previously reported [39], inhibition of actin assembly by cytB blocked the release of nascent phagosomes from the oral apparatus and prevented the accumulation of phagosomes in the cytoplasm (Figure 9C). Similarly, FM1–43 uptake, but not phagosome formation, appeared normal in cells treated for 5 min with the actin inhibitor latrunculin A (10 μM; data not shown). The results suggest that CME in *Tetrahymena* is less dependent on actin assembly than is phagocytosis.

### Localization of Drp1p Depends on a Novel Motif

**Figure 10:**
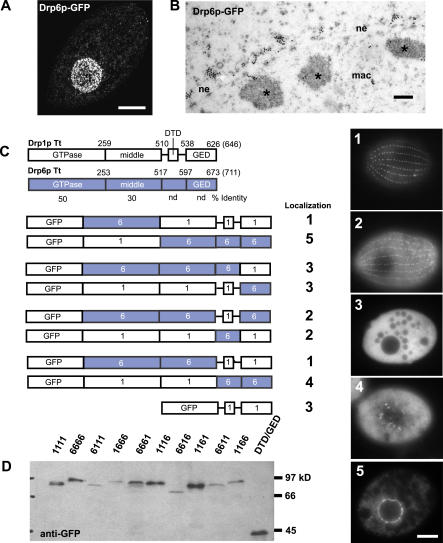
Analysis of Drp1p Targeting by Chimera Analysis (A) Compiled stack of confocal sections through fixed cell expressing Drp6p-GFP (driven by the *MTT1* promoter) shows localization at the nuclear envelope. Bar = 10 μm. (B) Immuno-gold visualization of Drp6p-GFP shows localization in clusters on the cytoplasmic face of the nuclear envelope (ne). Regions of heterochromatin within the macronucleus (mac) are indicated (*). Bar = 200 nm. (C) Domain comparison of Drp1p and Drp6p and diagrams of Drp-GFP chimeras (expressed under the *MTT1* promoter) indicating localization. Numbered images at right show localization patterns observed in cells expressing chimeric proteins. Bar = 10 μm. nd, not detected. (D) Western blot of total cell lysates from Drp-GFP cells confirms stability of full-length chimeras.

If classical dynamin and Drp1p are independently adapted to roles in endocytosis, the mechanisms of targeting to endocytic sites may be different. That the mechanisms are different is suggested by the sequence differences between the two proteins. The PH domain of classical dynamin binds phosphatidylinositol-4,5-P_2_ at the plasma membrane, which contributes to its localization [18,40]. At the position of the PH domain, *Tetrahymena* Drp1p has a short stretch of 28 residues with no recognizable motif. To ask which regions of Drp1p are important for localization, we created full-length chimeras between *DRP1* and a paralog whose product showed a different subcellular localization. An ideal partner for chimera construction was found after GFP-tagging one of the seven additional *Tetrahymena* DRPs. Drp6p-GFP localized at the nuclear envelope in a pattern easily distinguished from Drp1p-GFP (Figure 10A and 10B). These paralogs share 50% and 30% identity between the GTPase and middle domains, respectively, but none between the more C-terminal domains. Only one other dynamin (MxB, in humans) has previously been reported at the nuclear envelope [41].

We exchanged the coding sequence for several domains between *DRP1* and *DRP6* to create a series of chimeras, whose localization is shown in Figure 10C and stability is demonstrated in Figure 10D. Neither GTPase domain altered the localization of the remaining three domains (Figure 10D, 6111 and 1666). Exchanging the domain just C-terminal to the middle domain, in contrast, was highly informative. We named this domain the Drp targeting determinant (DTD). A chimera in which a Drp6p backbone contained the DTD of Drp1p (Figure 10D, 6616) was targeted to endocytic sites. Some of the chimera, however, was diffusely distributed in the cytoplasm, suggesting that targeting determinants also lay in other domains. We therefore tested chimeras in which both the DTD and GED were exchanged. Chimeras containing the DTD and GED of Drp1p strongly localized to coated pits (Figure 10D, 6611). The Drp1p GED was necessary for full targeting, but not sufficient, because exchanging just the GED between Drp1p and Drp6p resulted in only diffuse cytoplasmic localization (Figure 10D, 6661 and 1116). Similarly, although the combined DTD and GED were sufficient to define targeting in the context of the full-length protein, a construct consisting of just the Drp1p DTD and GED, linked to GFP, localized diffusely in the cytoplasm (Figure 10D, *xx*11). This may be explained if efficient localization requires oligomer assembly, for which at least three dynamin domains are required [42].

Interestingly, targeting of the reciprocal chimeras (Drp6p determinants in a Drp1p context) was significantly less efficient, and both the Drp6p DTD and GED were required for nuclear targeting (Figure 10D, 1166).

## Discussion

The molecular mechanisms underlying eukaryotic membrane traffic are likely to have an ancient origin because components of these pathways are conserved across multiple lineages. For example, phylogenetic analysis of syntaxins, a family of SNARE proteins involved in vesicle fusion, supported an early origin for the functional diversification of these paralogs, which implies a set of primitively differentiated pathways in membrane traffic [43,44]. Nonetheless, conclusions based on phylogenetics without accompanying functional data are limited, and potentially misleading, because genes within families can evolve new roles in individual lineages. The great majority of functional studies of membrane traffic have been performed in either animal cells or budding yeast. Because the fungal lineage diverged relatively recently from that of animals, when compared to many other eukaryotic lineages, the oft-cited interval “from yeast to humans” represents a surprisingly small sampling of the potential molecular variation among existing organisms [45]. Fortunately, an increasing number of species are associated with extensive genomic data and tools for gene manipulation, allowing functional analysis of genes within highly conserved families. We have used the combination of tools available in *Tetrahymena* to analyze the molecular machinery at endocytic structures called parasomal sacs.

### Clathrin-Mediated Endocytosis in *Tetrahymena*

Our analysis demonstrates that parasomal sacs are sites of clathrin-dependent membrane internalization. FM1–43 uptake was blocked by inducible expression of a domain of *Tetrahymena* clathrin, equivalent to the dominant negative hub domain of mammalian clathrin. In animal cells, this domain does not itself localize to coated pits [46], but the equivalent *Tetrahymena* domain localized to endocytic sites, suggesting that some mechanisms used for clathrin localization may differ. However, clathrin recruitment in *Tetrahymena,* as in animal cells, is likely to involve the AP-2 complex, because the μ subunit localized to the region of endocytic sites. Phylogenetic analysis of AP complexes, from *Tetrahymena* to humans, indicates that gene duplication and pathway-specific differentiation within this family predated the divergence of ciliates from higher eukaryotes.

Surprisingly, CME in *Tetrahymena* requires a DRP, Drp1p, which also localizes to endocytic sites. The function of dynamin in endocytosis was first recognized in *Drosophila* [47], but no comparable direct role has previously been demonstrated in any unicellular organism, nor for any DRP. The discovery of Drp1p's role in endocytosis in *Tetrahymena* therefore raises both evolutionary and mechanistic questions.

### Evolution of Dynamin-Related Proteins

The most widely conserved role for DRPs is in mitochondrial maintenance, and a DRP with that function has therefore been proposed as a possible founder of the dynamin family [15]. The association with endocytosis in metazoans may have involved subsequent gene duplication and neofunctionalization in that lineage. The endocytic function of Drp1p in *Tetrahymena* raises the possibility that endocytosis was a more ancestral function for dynamins, which was retained in metazoans and ciliates but lost in representatives from at least three other lineages, i.e., Fungi, trypanosomes, and red algae. This scenario was not, however, supported by our extensive phylogenetic analysis. Classical metazoan dynamins associate in a clade distinct from Drp1p. This was true whether the whole proteins, or individual domains, were compared, which argues against the idea that clustering may reflect lineage-specific partial gene conversion. Similarly, Drp1p was more related to a cornucopia of DRPs in both ciliates and *Plasmodium,* than to any metazoan protein. This clustering according to lineage, rather than according to function, suggests that dynamins independently underwent duplication and neofunctionalization in the Alveolates, a family including ciliates and *Plasmodium.* We therefore hypothesize that the functional similarity between classical dynamins and Drp1p reflects independent innovations, within the same protein family, in two distant lineages. Many DRPs may be fundamentally similar in their mechanism of action, namely, membrane deformation upon protein self-assembly, so one important class of innovations may be accounted for simply by mutation-induced changes in subcellular targeting.

Our discovery may not represent a unique example in the evolution of the dynamin family. Both *T. thermophila* Drp6p and *Homo sapiens* MxB are targeted to nuclear pores, but the functional relationship between the two proteins remains to be investigated. Moreover, the phylogenetic relationship is also ambiguous, because both are at the ends of long branches in a relatively bare region of the tree where some important branch nodes have intermediate bootstrap values. However, the phylogeny may become more robust as more protist genomes are sequenced. Future analysis may therefore reveal that Drp6p and MxB were independently recruited to play similar roles. Another potential example comes from *VPS1,* a *S. cerevisiae* DRP involved in vacuolar protein sorting, which is phylogenetically linked with mitochondrial DRPs (see Figure 8). However, interpreting the phylogeny of *VPS1* is complicated because both it and mammalian *DLP1,* a “mitochondrial” DRP, also function in peroxisomal division [48–50]. Additionally, no mammalian dynamin or DRP has been clearly demonstrated to be functionally equivalent to *VPS1.* In this case, therefore, the issue of functional convergence is unresolved.

An unresolved issue, not accounted for in the phylogenetic analysis, is the existence of classical dynamins with PRDs and PH domains in *Arabidopsis.* However, their relationship to metazoan dynamins is currently ambiguous, because the plant proteins are strikingly diverged in sequence, including within the otherwise strictly conserved catalytic GTPase motifs, and their roles are not yet clear [21]. Another informative lineage may be that of *Dictyostelium discoideum,* where disruption of a *DRP* gene resulted in pleiotropic defects in organelle morphology, cytokinesis, and endocytosis [51].

### Mechanism of Dynamin Function in Endocytosis

The role of Drp1p in endocytosis was also unexpected because the *Tetrahymena* protein lacks two domains important for the activity of classical dynamin. The absence of a PRD in Drp1p suggested that Drp1p's function does not depend upon actin recruitment. While essential for endocytosis in *S. cerevisiae,* actin appears to have a less critical role in animal cells [35], although recent work suggests some role for actin at several stages of vesicle formation [52]. In *Tetrahymena,* neither cytB nor latrunculin A, at levels that completely blocked phagocytosis, had a discernible effect upon FM1–43 internalization. We note, however, that this organism appears to encode at least four actin genes, whose sequences are highly divergent for such an otherwise highly conserved protein (unpublished data). Therefore, we cannot rule out that some actin isoforms, resistant to these agents, may be involved.

Also missing in Drp1p, but present in classical dynamins, is the PH domain. To determine how Drp1p is targeted in its absence, we characterized additional *Tetrahymena DRP* family members to identify a paralog suitable for constructing informative chimeras. The *Tetrahymena* genome contains eight *DRP* genes, an unusually large number when compared to other unicellular organisms. Similarly, the genome of *Paramecium* appears to contain nine *DRP* genes. The apparent lineage-specific expansion of this gene family may reflect the elaborate endomembrane system of ciliates [53]. To our surprise, a GFP-tagged DRP in *Tetrahymena,* Drp6p, targeted to the nuclear envelope. This distinct localization combined with structural similarity provided us with a partner for chimera analysis.

Analysis of a set of *DRP1*-*DRP6* chimeras demonstrated that localization depended on the two C-terminal domains of the protein. The more important was the DTD, because this domain by itself gave partial localization. Based on its location in the primary sequence, the DTD is likely to occupy the same position as the PH domain in classical dynamin, also involved in localization. The DTD shows no sequence similarity to a PH domain, however. It is unlikely to represent a functional PH domain diverged to the point of being unrecognizable, because highly conserved PH domains are easily recognized in other *Tetrahymena* genes (unpublished data). These observations are consistent with the hypothesis of independent innovation of ciliate and metazoan endocytic dynamins but are challenging to reconcile with a model based on shared derivation and multiple losses in other lineages.

Our results on targeting determinants are similar, but not identical, to those from a similar domain-swapping approach in a study of dynamin-2 and a mitochondrial DRP, *DLP1,* in mammalian cells [54]. In that study, DLP1 targeting required determinants in the C-terminal domains but also in the middle domain. Because the middle domain is involved in self-assembly, this requirement suggested that localization involves motifs whose affinity is too low, in the monomeric state, for effective targeting. Consistent with this idea, we found that the DTDs and GEDs of Drp1p were targeted to endocytic sites only in the context of the holo-protein. However, and unlike DLP1, targeting was efficient even if the middle domain came from Drp6p. One possibility is that the chimera containing the Drp6p middle domain in a Drp1p backbone undergoes efficient self-assembly, in a way that is not possible for the corresponding domains in *DLP1* and dynamin-2. This difference in interchangeability is not, however, supported by the sequences themselves, because Drp1p and Drp6p appear more unrelated than *DLP1* and dynamin-2. A second possibility is that Drp1p targeting does not require self-assembly. This issue, as well as the mechanism of targeting, will be clarified in future studies.

### Parasomal Sacs and Ciliate Physiology

Our results suggest that CME in *Tetrahymena* is essential, and future work may identify the physiological cargoes in this pathway. The proximity of parasomal sacs to basal bodies, and the presence of AP-2, suggests the possibility that these sites are adapted for uptake of sensory receptors on cilia, either for turnover or for signaling from endosomes [55]. Complete inhibition of visible FM1–43 uptake, in cells expressing clathrin dominant negative constructs, is consistent with the idea that no compensatory pathways of clathrin-independent internalization exist at these sites or elsewhere on the cell surface, although we cannot rule out the possibility of dominant negative inhibition of a parallel pathway that utilizes some common machinery.

In conclusion, our study reveals unexpected relationships, both molecular and evolutionary, between endocytosis in ciliates and animals. This includes the sharing of some features that are absent in fungi, even though the fungal and animal lineages are much more closely related than either is to ciliates [45]. The tools available in *Tetrahymena* [56] should facilitate the discovery and analysis of regulatory features of the endocytic pathway mediated by clathrin and Drp1p. Whether convergent evolution of function has occurred within the dynamin family, and how frequently, may be further illuminated by analysis of the large family of DRPs in ciliates.

## Materials and Methods

### *Tetrahymena* strains and culture conditions.

Wild-type B2086, CU427, and CU428.1 strains of *T. thermophila* were provided by Peter Bruns (Cornell University). Strain B*VI was provided by Sally Allen (University of Michigan). Unless stated otherwise, cells were grown at 30 °C in SPP medium (1% proteose peptone, 0.2% dextrose, 0.1% yeast extract, 0.009% ferric EDTA). For conjugation, cells of different mating types growing in log-phase were washed, starved (16–20 h at 30 °C), and mixed in DMC, a one-tenth dilution of Dryl's (1.7 mM sodium citrate, 1 mM NaH_2_PO_4_, 1 mM Na_2_HPO_4_, 1.5 mM CaCl_2_) supplemented with an additional 0.1 mM MgCl_2_ and 0.5 mM CaCl_2_.

### CHC hub, AP μ subunits, and dynamin-related genes.

A single, CHC homolog *(CHC1)* was identified from whole genome sequence of *Tetrahymena* macronuclear DNA (preliminary sequence data were obtained from The Institute for Genomic Research Web site at http://www.tigr.org). Using primers designed from genomic sequence, the 3′ portion of the gene encoding the hub fragment was amplified by PCR using a cDNA library from growing *Tetrahymena* [57]. AP-1 μ subunit *(APM1A),* AP-2 μ subunit *(APM2),* and a dynamin-related *(DRP1)* homolog were identified from ESTs (unpublished data) and full-length clones were amplified by PCR. An additional AP-1 μ subunit gene *(APM1B),* a putative AP-4 μ subunit homolog (*APM4),* and seven additional *DRP* genes *(DRP2* through *DRP8)* were identified from genome sequence. Full-length *DRP6* was amplified from genomic DNA. All PCRs were performed with Pfu-Ultra Taq polymerase (Stratagene, La Jolla, California, United States). Products were cloned into pCRII (Invitrogen, Carlsbad, California, United States) and sequenced to confirm accuracy.

### Phylogenetic analysis.

Dynamin domains were defined using the NCBI Conserved Domains database (http://www.ncbi.nlm.nih.gov/Structure/cdd/cdd.shtml) and were aligned using ClustalW (http://www.ebi.ac.uk/clustalw/), as were AP μ subunit sequences. Unambiguously aligned regions of sequence were identified by eye and used for analysis. Protein maximum likelihood analysis was done using PROML (Phylip 3.6; Joe Felsenstein, University of Washington). The topology shown is the best tree generated by PROML. Bootstrapping analysis by maximum likelihood and maximum parsimony using PROTPARS was performed with 100 datasets. All alignments are available upon request.

### Construction of *DRP1* germline knockout strains.

The *neo3* cassette [27] was ligated into a genomic clone of *DRP1* at EcoRI sites to generate the *drp1::neo3* construct (see Figure 5A). B2086 and CU428 cells were mixed for 2.5 h and transformed with linearized *drp1::neo3* by biolistic particle bombardment to target *DRP1* in the micronucleus by homologous recombination. Knockout heterokaryon strains with disrupted *DRP1* in the micronucleus and wild-type copies of *DRP1* in the somatic macronucleus were isolated as described [58]. Subsequent matings of the germline knockout strains resulted in drug-resistant progeny with disrupted *DRP1* in both nuclei.

### Construction and expression of transgenes.

Two constructs were designed to rescue the progeny of *DRP1* germline knockout matings. A *DRP1-HA* construct was built by ligating the HA epitope sequence into PmeI and XhoI sites upstream of a modified *DRP1* genomic clone. The *DRP1-MTT1* construct was built by replacing the *MTT1* gene with a cDNA clone of *DRP1* in a plasmid containing the *MTT1* locus [27]. Knockout heterokaryons were mixed for 24 h and transformed with these linearized constructs by biolistic bombardment. Drug-resistant clones were recovered and, in the case of the *DRP1-MTT1*-expressing cells, maintained in 1 μg/ml cadmium. An additional strain was derived from the *DRP1-HA* line, by transforming it biolistically with the *APM2-GFP* fusion. For this transformation, the *APM2-GFP* fusion was engineered into a vector (nCV-B) that permits selection with Blasticidin (60 μg/ml) [59].

Other monomeric eGFP (GFP) fusions and dominant negative *DRP1* alleles resided in a modified version of the rDNA expression vector pVGF (Meng-Chao Yao, FHCRC, Seattle, Washington, United States). Genes were inserted downstream of the inducible *MTT1* promoter and GFP with a ligation into XhoI and ApaI sites. *APM1A, APM1B, APM2, DRP1, DRP6,* and the hub portion of *CHC1* were each amplified by PCR with flanking XhoI and ApaI sites. Chimeras between *DRP1* and *DRP6* were assembled by an overlap PCR strategy [60] with domain borders defined by the NCBI Conserved Domains database and refined by eye to match between *DRP1* and *DRP6*. The dominant negative alleles *DRP1-K51E* and *DRP1-T72F* were generated by site-directed mutagenesis PCR (Stratagene) and ligated into pVGF via PmeI and ApaI sites that excluded GFP from the constructs. B2086 and CU428 cells were mixed for 10 h and transformed with plasmid by electroporation [61]. To bring transgenes into expression, cells were treated with 1 μg/ml cadmium for 12 to 16 h, unless otherwise noted. A complete description of the strains used in this study is found in Table S1.

### Uptake assays.

All assays were performed with log-phase growing cells starved for 2 h in DMC. Cells were treated with 5 μM FM1–43 (Molecular Probes, Eugene, Oregon, United States) and placed under coverslips for immediate viewing. For time-course assays, cells were treated with FM1–43 for 5 min, washed three times in DMC, and immobilized using a rotocompressor (made by Warren Ringlien, Carleton College, Northfield, Minnesota, United States) for observation over a time course.

FM1–43 uptake comparisons between strains were performed by treating CU428 with 200 ng/ml DAPI in DMC for 5 min. Cells were washed and mixed with an equal number of cells expressing either the *CHC1* hub fragment or the *K51E* allele of *DRP1* after 3 h in 1 μg/ml cadmium. The same mixing protocol was used for cells treated with 25 μM cytB for 30 min or 10 μM latrunculin A for 5 min, or *DRP1-MTT1* cells maintained without cadmium for 16 h. All cell mixtures were treated with 10 μM FM1–43 for 5 min, washed three times, and photographed after 10 min. For experiments with cytB, the drug was also included during the FM1–43 incubation in some trials.

To quantify uptake, 20 representative images per experiment were analyzed on a Macintosh computer using NIH Image 1.63 (http://rsb.info.nih.gov/nih-image/). The images chosen were those in which both a control cell and an experimental cell were captured in a single frame. Mean density measurements from each set of cells were averaged for comparison. To assess phagocytic uptake in cytB-treated cells, 0.1% India ink was substituted for FM1–43.

### Immunocytochemistry and fluorescence microscopy.

Cells were fixed and prepared as described previously [62] but with incubations performed at room temperature. Basal bodies were visualized using a 1:1,000 dilution of monoclonal antibody 20H5, recognizing centrin (provided by Jeff Salisbury, Mayo Clinic, Rochester, Minnesota, United States), followed by 1% (v/v) Texas red-conjugated goat anti-mouse antibody (Jackson ImmunoResearch, West Grove, Pennsylvania, United States). Similarly, Drp1p-HA was visualized using a 1:1,000 dilution of monoclonal HA.11 (Covance, Princeton, New Jersey, United States). For two-color labeling, GFP fluorescence was enhanced by including 0.5% (v/v) rabbit anti-GFP primary antibodies (Molecular Probes), followed by 1% (v/v) fluorescein-conjugated anti-rabbit antibody (Jackson ImmunoResearch). This also guaranteed detection of the entire pool of GFP-labeled protein. Samples were viewed under a Zeiss (Thornwood, New York, United States) Axiovert microscope interfaced with a Zeiss LSM 510 confocal laser system and software. Endocytic uptake assays were observed using a Zeiss Axioplan 2 microscope interfaced with a Zeiss Axiocam and Axiovision software.

### Cryofixation and immunoelectron microscopy.

Cells were grown in SPP with 150 mM mannitol and 2 μg/ml CdCl_2_. After cells were collected via centrifugation, they were washed with SPP and mannitol, and a few microliters of the cell pellet/slurry were transferred to an aluminum planchette (Type A) with a 100-μm-deep well (engineering office of M. Wohlwend, Senwald, Switzerland) and sandwiched with the flat side of a Type B aluminum planchette, coated with hexadecene [63]. Cells were cryofixed in a BAL-TEC (Balzers, Switzerland) HPM-010 high-pressure freezer and then freeze-substituted in 0.25% glutaraldehyde and 0.1% uranyl acetate in acetone. Embedding was as described by Giddings [63] except that isopropyl alcohol was used in place of methanol to maintain the sample at −45 °C for polymerization of the HM20.

Embedded cells were serially sectioned (50–60 nm) and put on formvar-coated nickel grids. Some sections were immunolabeled with a polyclonal anti-GFP antibody diluted 1:200 in a blocking solution of 1% nonfat dry milk in PBST and 15 nm of colloidal gold-conjugated secondary antibodies (Ted Pella, Redding, California, United States). Samples were stained with 2% uranyl acetate in 70% methanol/30% water for 5 min and lead citrate for 4 min. The sections were viewed on a Philips (Eindhoven, Netherlands) CM10 electron microscope operating at 80 kV. Images were captured with a Gatan (Pleasanton, California, United States) digital camera and viewed with the Digital Micrograph Software package (Gatan).

### Blotting.

For Southern analysis, genomic DNA was prepared from CU428-, *DRP1-MTT1-,* and *DRP1-HA*-expressing cells and detected with a probe corresponding to the 5′ EcoRI restriction fragment of *DRP1.* Northern blotting of total RNA extracted from CU428- and *DRP1-MTT1-*expressing cells maintained with and without cadmium, and *DRP1-HA-* expressing cells was performed following standard techniques [64] using a *DRP1* riboprobe. A cross-reactive band near 1 kb serves as a loading control. For Western analysis, whole-cell lysates were prepared as described previously [62]. GFP-tagged chimeras of Drp1p and Drp6p were detected using 0.125% (v/v) rabbit anti-GFP primary antibodies (Molecular Probes), followed by 0.1% (v/v) AP-conjugated anti-rabbit antibody (Jackson ImmunoResearch).

## Supporting Information

Figure S1 Alignment of DRPs from *Tetrahymena* with Dynamin-1 *(H. sapiens)*
The peptide sequence alignment was generated using ClustalX and highlights residues that are completely conserved (*), strongly conserved (:), and weakly conserved (.). The color coding indicates which residues are small/hydrophobic (blue), acidic (purple), basic (red), hydroxyl/amine (green), glycines (brown), and prolines (yellow). Also indicated are domain boundaries defined using the NCBI Conserved Domains database, the strictly conserved catalytic motifs in the GTPase domain, and the residues where substitutions generated dominant negative alleles of *DRP1* (arrowheads).
(1.4 MB DOC)

Figure S2 Expression of *DRP4-GFP* and *DRP7-GFP*
(A) Confocal sections of a live cell expressing *DRP7-GFP* (under the *MTT1* promoter) are shown at the cortex, i.e., just beneath the cell surface (left), and at the middle of the cell (middle). Most GFP signal is at the cortex.
(B) A confocal section from the middle of a live cell expressing *DRP4-GFP* (under the *MTT1* promoter) showing that punctate GFP fluorescence is distributed throughout the cytoplasm (right). Bar = 5μm.
(C) *DRP7-GFP*-expressing cells were incubated for 60 min in 50 nM MitoTracker Red CMXRos (Molecular Probes). Shown is a cortical confocal section of a living cell, revealing association of Drp7p-GFP with mitochondria. Bars = 5 μm.
(21.3 MB EPS)

Figure S3 Phylogenetic Tree of Dynamin and DRPs from a Bayesian Approach The alignment used for maximum likelihood and maximum parsimony analysis in Figure 6 was subjected to Bayesian-based tree construction using MrBayes v3.0 [65]. After the burn-in phase, every 100th sample of 1 × 10^6^ generations was considered. The resulting 50% majority rule consensus tree is shown with percent posterior probabilities indicated at each node.
(168 KB EPS)

Table S1 Strains Used in This Study (32 KB DOC)

### Accession Numbers

The *Tetrahymena* and *Paramecium* genes discussed in this paper have been submitted to GenBank (http://www.ncbi.nlm.nih.gov/Genbank/) under the following accession numbers: *Tetrahymena* APM1A (DQ219841), APM1B (DQ219842), APM2 (DQ219843), CHC1_hub (DQ219844), DRP1 (DQ219845), DRP4 (DQ219846), DRP6 (DQ219847), and DRP7 (DQ219848); and *Paramecium* DRPA1 (CR856028), DRPA2 (CR856027), DRPB1 (CR856026), DRPB2 (CR856025), DRPC1 (CR856024), DRPC2 (CR856023), DRPD (CR856022), DRPE (CR856021), and DRPF (CR856020).

## Abbreviations

μ subunit: adaptor protein medium subunit
AP: adaptor protein
CHC: clathrin heavy chain
CME: clathrin-mediated endocytosis
cytB: cytochalasin B
DRP: dynamin-related protein
DTD: Drp1 targeting determinant
GED: GTPase effector domain
GFP: green fluorescent protein
PH: pleckstrin homology
PRD: proline-rich domain
