# Supplementary material for: Elucidation of Clathrin-Mediated Endocytosis in Tetrahymena Reveals an Evolutionarily Convergent Recruitment of Dynamin
Source: PLoS Genet. 2005 Nov 4;1(5):e52. doi: 10.1371/journal.pgen.0010052 (PMC1277907; doi:10.1371/journal.pgen.0010052)
Supplement: Supplementary file 1 [file pgen.0010052.sg001.doc]

**Supplemental Figure 1**
