# Supplementary material for: Elucidation of Clathrin-Mediated Endocytosis in Tetrahymena Reveals an Evolutionarily Convergent Recruitment of Dynamin
Source: PLoS Genet. 2005 Nov 4;1(5):e52. doi: 10.1371/journal.pgen.0010052 (PMC1277907; doi:10.1371/journal.pgen.0010052)
Supplement: Supplementary file 2 [file pgen.0010052.st001.doc]

**Supplemental Table 1**. Strains used in this study.

| **Strain** | **Mic genotype** | | **Mac genotype** | **Mac phenotype** | |
| --- | --- | --- | --- | --- | --- |
| B2086 | WT | | WT | II | |
| CU427 | *chx1-1/chx1-1* | | WT | cy-s, VI | |
| CU428 | *mpr1-1/mpr1-1* | | WT | mp-s, VII | |
| B*VI | nonfunctional mic | | WT | VI | |
| UC410  (DRP1-KO1) | *drp1-1[Δ_1−724_::neo3] / drp1-1[Δ_1−724_::neo3]* | | WT | pm-s, ? | |
| UC411  (DRP1-KO2) | *drp1-1[Δ_1−724_::neo3] / drp1-1[Δ_1−724_::neo3]* | | WT | pm-s, ? | |
| UC412  (DRP1-MTT1) | *drp1-1[Δ_1−724_::neo3] / drp1-1[Δ_1−724_::neo3]* | | *drp1-1[Δ_1−724_::neo3], mtt1-1[Δ::DRP1]* | pm-r, ? | |
| UC413  (DRP1-HA) | *drp1-1[Δ_1−724_::neo3] / drp1-1[Δ_1−724_::neo3]* | | *drp1-1[Δ_1−724_::neo3],* *drp1-2[HA_N_]* | pm-r, ? | |
| UC431  (DRP1-HA, APM2-GFP) | *drp1-1[Δ_1−724_::neo3] / drp1-1[Δ_1−724_::neo3]* | | *drp1-1[Δ_1−724_::neo3],* *drp1-2[HA_N_ ], apm2-1 [MTT1^P^, GFP_N_ ]* | pm-r, blast-r, ? | |
| The following strains express transgenes from the rDNA vector pVGF | | | | | |
| **Strain** | **Mic genotype** | **Mac genotype** | | | **Mac phenotype** |
| UC414  (CHC1-HUB) | WT | *chc1-1[Δ_1−1083_ MTT1^P^]* | | | pm-r, ? |
| UC415  (CHC1-HUB-GFP) | WT | *chc1-2[Δ_1−1083_ MTT1^P^, GFP_N_]*, | | | pm-r, ? |
| UC416  (APM2-GFP) | WT | *apm2-1[MTT1^P^, GFP_N_]* | | | pm-r, ? |
| UC417  (APM1A-GFP) | WT | *apm1A-1[MTT1^P^, GFP_N_]* | | | pm-r, ? |
| UC418  (APM1B-GFP) | WT | *apm1B-1[MTT1^P^, GFP_N_]* | | | pm-r, ? |
| UC419  (DRP1-K51E) | WT | *drp1-3[MTT1^P^, K51E]* | | | pm-r, ? |
| UC420  (DRP1-GFP) | WT | *drp1-4[MTT1^P^, GFP_N_]* | | | pm-r, ? |
| UC421  (DRP6-GFP) | WT | *drp6-1[MTT1^P^, GFP_N_]* | | | pm-r, ? |
| UC422  (DRP1116-GFP) | WT | *drp1-5[Δ_538−645_::drp6_582−711_ MTT1^P^, GFP_N_]*, | | | pm-r, ? |
| UC423  (DRP1166-GFP) | WT | *drp1-6[Δ_510−645_::drp6_517−711_ MTT1^P^, GFP_N_]* | | | pm-r, ? |
| UC424  (DRP1666-GFP) | WT | *drp1-7[Δ_259−645_::drp6_253−711_ MTT1^P^, GFP_N_]* | | | pm-r, ? |
| UC425  (DRP1161-GFP) | WT | *drp1-8[Δ_510−538_::drp6_517−582_ MTT1^P^, GFP_N_]* | | | pm-r, ? |
| UC426  (DRP1C2-GFP) | WT | *drp1-9[Δ_1−510_ MTT1^P^, GFP_N_]* | | | pm-r, ? |
| UC427  (DRP6661-GFP) | WT | *drp6-2 [Δ_582−711_::drp1_538−645_  MTT1^P^, GFP_N_]* | | | pm-r, ? |
| UC428  (DRP6611-GFP) | WT | *drp6-3 [Δ_517−711_::drp1_510−645_  MTT1^P^, GFP_N_]* | | | pm-r, ? |
| UC429  (DRP6111-GFP) | WT | *drp6-4 [Δ_253−711_::drp1_259−645_  MTT1^P^, GFP_N_]* | | | pm-r, ? |
| UC430  (DRP6616-GFP) | WT | *drp6-5 [Δ_517−582_::drp1_510−538_  MTT1^P^, GFP_N_]* | | | pm-r, ? |

Micronucleus (Mic), Macronucleus (Mac), ? denotes unknown mating type.
